# Cytotoxicity and Bioimaging Study for NHDF and HeLa Cell Lines by Using Graphene Quantum Pins

**DOI:** 10.3390/nano10122550

**Published:** 2020-12-18

**Authors:** Seong-Beom Jeon, Monica Samal, Saravanan Govindaraju, Rupasree Ragini Das, Kyusik Yun

**Affiliations:** 1 Department of Bionanotechnology, Gachon University, Seongnam 13120, Korea or sbjeon0430@gist.ac.kr (S.-B.J.); biovijaysaran@gmail.com (S.G.); rupasree.ragini@gmail.com (R.R.D.); 2 School of Environmental and Science Engineering, Gwangju Institute of Science and Technology (GIST), Gwangju 61005, Korea; 3 Department of Material Science and Engineering, North Carolina State University, Raleigh, NC 27695, USA; ms.phd.sci@gmail.com

**Keywords:** graphene quantum pins, photoluminescence, cytotoxicity, cellular response, cellular distribution

## Abstract

Herein, we report the synthesis of an interesting graphene quantum material called “graphene quantum pins (GQPs)”. Morphological analysis revealed the interesting pin shape (width: ~10 nm, length: 50–100 nm) and spectral analysis elucidated the surface functional groups, structural features, energy levels, and photoluminescence properties (blue emission under 365 nm). The difference between the GQPs and graphene quantum dos (GQDs) isolated from the same reaction mixture as regards to their morphological, structural, and photoluminescence properties are also discussed along with the suggestion of a growth mechanism. Cytotoxicity and cellular responses including changes in biophysical and biomechanical properties were evaluated for possible biomedical applications of GQPs. The studies demonstrated the biocompatibility of GQPs even at a high concentration of 512 μg/mL. Our results suggest GQPs can be used as a potential bio-imaging agent with desired photoluminescence property and low cytotoxicity.

## 1. Introduction

In recent years, graphene-related applications have been extensively investigated in wide-ranging fields such as solar cell, field-effect transistor, LED, drug and gene delivery, and cell culture platform [1,2,3,4,5,6,7,8,9]. The various properties of graphene including zero band gap [10], excellent electron transfer ability [11], and mechanical strength [12] are attractive enough to inquire about their modifications and application perspectives. Quantum dots (QDs) also have been studied in several fields such as LED [13], solar cells [14], and bio-imaging agent [15] due to the quantum confinement effect, and their excellent photoluminescence properties [16]. However, these two materials have their drawbacks: Graphene is difficult to utilize in biomedical applications due to its non-homogeneous size and the problem of large scale synthesis. In addition, QDs are usually synthesized from heavy metals such as Cd and Ga, which can be hazardous to human being and environment [17,18,19,20]. Studies show that the organic quantum material called GQDs having a few carbon atom layers as graphene sheets and quantum confinement effect can overcome these two problems. Research fields such as delivery vector (protein, gene, and drug) [21,22,23,24], fluorescence probes for bio-imaging [25,26,27] are considered to have the potential applications to apply GQDs due to their high surface area, good biocompatibility, and excellent photoluminescence properties. The synthetic methods can be categorized in two ways: “Top-down” and “bottom-up” approaches. The top-down approach includes cutting carbon materials [28], electrochemical methods [29], oxygen plasma treatment [30], hydrothermal process [31], microwave-assisted methods [32], nanotomy assisted methods [33], and laser fragmentation [34]. Very attractive graphene nanomaterials which have different shapes with GQDs have been discovered, such as graphene nanoribbon [35] graphene quantum ring [36] and graphene onion [37]. Recently, the “bottom-up” approach is preferred due to the controllable size tunning, and easy surface modification of graphene nanomaterials and the prevention of excess acid during preparation. The “bottom-up” approach mainly involves solution chemistry with different seed materials such as adenosine triphosphate (ATP) [38], citric acid [39], and glucose, [40].

Herein, we first introduce a synthesis method of a new kind of graphene quantum materials called “graphene quantum pins (GQPs)” having pin shape via a “bottom-up” approach from glucose. The morphological and structural investigations confirmed the growth mechanism of GQPs. Besides, the cytotoxicity of GQPs in normal human dermal fibroblast (NHDF) and cervical cancer cells (HeLa) was tested. The morphological, biophysical, and biomechanical properties of the NHDF and HeLa cell lines were investigated by the Bio-AFM (Bruker Co., Billerica, MA, USA) and SEM (JEOL Ltd., Tokyo, Japan). The bio-imaging and cellular distribution were carried out with a fluorescence microscope and laser scanning confocal microscope. Given that, collected data show that GQPs have potential in the biomedical application as a bio-imaging agent. GQDs were also isolated from the same reaction mixture. The morphology, spectral analysis, and bio-studies of GQDs were compared with those GQPs.

Bio-Atomic force microscope (Bio-AFM) has been used as a valuable instrument to investigate the property of biological samples (e.g., live cell, extracellular matrix) [41,42]. Recently, the important function of Bio-AFM is underlined in the biomedical application which includes stem cell differentiation [43,44], and molecular–molecular interaction [45]. Hence, in the study of interface, the utilization of Bio-AFM has shown the interaction between cells and nanomaterials at pico Newton (pN) sensitivity and high accuracy. Up to now, little effort has been focused on the morphology of the cell, the biophysical (average cell height, cell spreading area, RMS roughness), and the biomechanical (cell stiffness) property before and after the nanomaterials treatment. So, an attempt is undertaken in this study to investigate the effect of GQPs on the physical/mechanical properties of cells.

## 2. Experimental Section

### 2.1. Synthesis and Characterization of GQPs

GQDs and GQPs were synthesized by carbonization and dehydration of glucose via the modified method of the previous report by J. Yang et al. [46]. Details of synthesis and characterization is provided in supporting information.

### 2.2. Cytotoxicity

NHDF (Cha University, Seongnam, Korea) and HeLa (Cha University, Seongnam, Korea) were cultured Dulbecco’s modified eagle medium (DMEM) (Thermo fisher scientific, Waltham, MA, USA), supplemented with 10% fetal bovine serum (FBS, Gibco, GE healthcare, Chicago, IL, USA) and antibiotics (100 U/mL penicillin and 100 μg/mL streptomycin, Thermo fisher scientific, Waltham, MA, USA), with high and low glucose concentrations, respectively. The cytotoxicity of GQDs and GQPs was measured by Cell Counting Kit-8 assay (CCK-8 assay, Dojindo, CK-04) according to the manufacturer’s instruction. NHDF and HeLa were cultured in 96 well plates at the seeding density of 5 × 10^3^/well and were incubated for 24 h in normal culture conditions (37 °C and 5% CO_2_). Typically, GQDs and GQPs solution was treated with DI water serial dilution ranging from 1024 μg/mL to 32 μg/mL. After the treatment of the samples, they were incubated for 24 h and washed with phosphate buffered saline (PBS) (Sigma Aldrich, St. Louis, MO, USA) 3 times for removing residual samples. Then, CCK-8 solution (10 μL/well) was added to each well and incubated for 1 h. A spectrometer (Synergy H1, Biotek instrument, Winooski, VT, USA) was used to measure absorbance at 450 nm. This study was repeated 3 times with triplicate.

### 2.3. Reactive Oxygen Species(ROS) Generation

For the measurement of ROS generation, 2′, 7′-Dichlorofluorescein diacetate (DCFH-DA) assay was carried out following a previous study [47]. NHDF and HeLa were seeded at the density of 10^4^ cells/well in black 96 well plate for 24 h. One hundred microliters of DCFH-DA in culture medium was added to each well and incubated at normal incubation condition for 30 min. After the suction of DCFH-DA, 200 μL of GQDs and GQPs solution (1024, 512, 256, 128, 64, and 32 μg/mL) was added to each well. To block light, the plates were covered with aluminum foil and placed in an incubator for 24 h. The intensity of the ROS probe was measured at 0 min and 24 h on a spectrometer (Synergy H1, BioTek) with excitation wavelength at 485 nm, absorbance at 538 nm, and a peak at 530 nm. We assumed that the ROS generation level of control cell is 0 theoretically and estimated the relative ROS generation of cells treated with GQDs and GQPs of various concentrations. The results are adduced as the average ROS intensity at 24 h subtracted by 0 min background intensity from three experiments with triplicate.

### 2.4. AFM and SEM Imaging of Cells

For AFM imaging, 0.2% gelatin was coated on 18 mm glass coverslips. Then, NHDF and HeLa were cultured at a seeding cell density of 5 × 10^4^ and incubated with 512 μg/mL GQDs and GQPs for 24 h. After 24 h, the medium was removed, and samples were washed 3 times with PBS. The live-cell images were taken in liquid contact mode using the DNP-10 silicon nitride AFM probe (Bruker Co., Billerica, MA, USA). The whole-cell, the nuclei, and the cytoplasm were taken, and the scanning size was adjusted to 100 μm × 100 μm, 40 μm × 40 μm, 10 μm × 10 μm following the sequence to provide the typical surface features while maintaining high resolution. For SEM imaging, the cells were passed through the cell fixation with 2% glutaraldehyde (Sigma Aldrich, St. Louis, MO, USA) and 4% paraformaldehyde (Sigma Aldrich, St. Louis, MO, USA), in turn. Then, the cells were washed serially with diluted (60%, 70%, 80%, 90%, and 99%) ethanol (Sigma Aldrich, St. Louis, MO, USA). After the dehydration of the cell, the samples were coated with platinum, and SEM images were taken in 3 regions as mentioned in the AFM study.

### 2.5. Cellular Response

AFM force spectroscopy was carried out following our previous study [48,49]. NHDF and HeLa cells were cultured as described in AFM imaging. Bio-AFM (JPK instrument, Bruker Co., Billerica, MA, USA) was used for the analysis of the biophysical (cell height, roughness, and spreading area) and biomechanical change (cell stiffness; Young’s modulus). A CONT-S sphere probe (5 μm radius, Nanoworld services GmbH, Erlangen, Germany) with a 0.4 N/m force constant was used to measure cell stiffness which refers to young’s modulus. After scanning briefly, the region of interest, 25 spots of the grid were checked for cell stiffness. The ramp size and the loading speed were adjusted to 1 μm and 1 μm/s. To prevent cell surface defects and Hertz model limitation, a series of indentation forces (0.5 nN~1.0 nN) were tested to calibrate the indentation depth in the range of 0.5 to 1.5 μm. Tip–sample separation curves and the Young’s modulus were determined via Hertz’s contact model using JPK data processing software (Bruker Co., Billerica, MA, USA). The Poisson’s ration was set to 0.5. For cell height and roughness, 2D AFM image was used to calculate via JPK image processing software. ImageJ software (National Institute of Health, Bethesda, MD, USA) was used for the cell spreading area.

### 2.6. Bio-Imaging and Cellular Distribution

In the bio-imaging study, both cells were treated with GQDs and GQPs solutions (512 μg/mL) at 37 °C for 2 h, 6 h, 12 h, and 24 h, respectively. After incubation, the cells were washed with PBS 3 times and imaged using the fluorescence microscopy (IRISTM Digital Cell Imaging System, Logos Biosystems, Anyang, Korea). GFP (excitation: 470 nm/emission: 530 nm) LED filter cube (Logos Biosystems, Anyang, Korea) was used to observe the fluorescence image. In cellular distribution, the cell culture condition was the same as the bio-imaging study. Besides, the cells were incubated at normal culture condition for 24 h and transferred and maintained at 4 °C followed by incubation with samples for 6 h, to investigate the mechanism of penetration of GQDs and GQPs. After washing with PBS 3 times, the confocal image of the cells was taken by using the laser scanning confocal microscope (Nikon PCM 2000, Tokyo, Japan) under 488 nm excitation wavelength and 530 nm filter.

### 2.7. Statistical Analysis

The data of CCK-8 assay, ROS generation measurement, and cellular response were presented as average and standard deviations. An unpaired student *t*-test was used to analyze the difference between the control groups and the experimental groups. For all analyses, the probability of Type-I error ≤0.05 was considered statistically significant. These results were obtained from three experiments, each analyzed in triplicate.

## 3. Results and Discussion

### 3.1. Growth Mechanism and Characterization of GQPs

**Figure 1:**
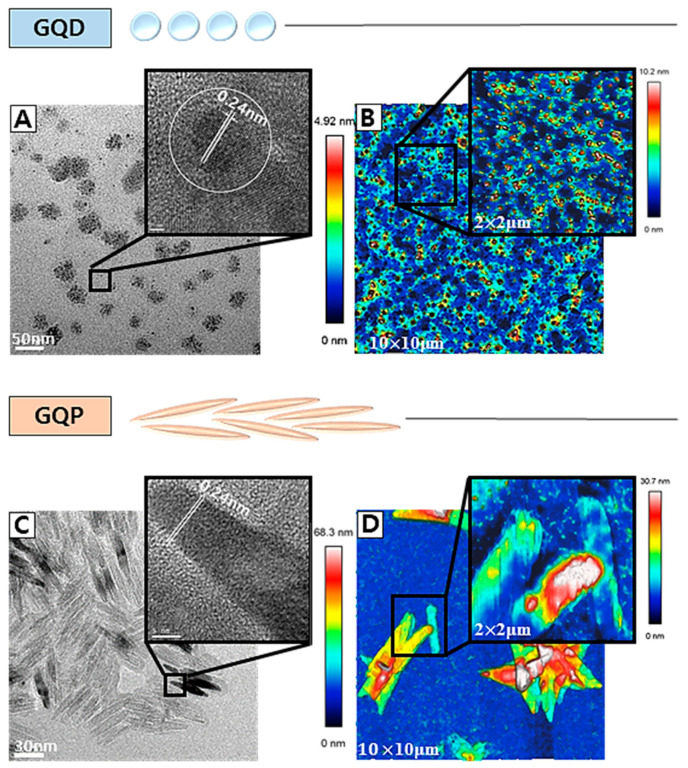
HR-TEM image and AFM image of (**A**,**B**) graphene quantum dos (GQDs) and (**C**,**D**) Graphene Quantum Pins (GQPs). The scale bars, 50 nm (**A**,**C**), 5 nm (inset). HR-TEM images are shown in inset image. (**B**,**D**) The AFM image show some aggregation of GQDs and GQPs due to electrostatic attraction. The maximum heights are 4.92 nm, 68.3 nm for GQDs and GQPs, respectively.

**Scheme 1:**
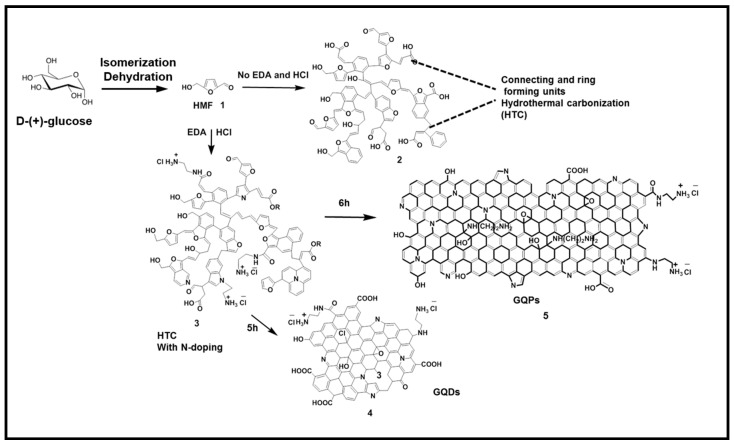
The growth mechanism of GQPs from glucose.

GQDs and GQPs were synthesized successfully using dehydration of carbonization of D-(+)-glucose by a hydrothermal process and the mechanism is given in Scheme 1. Diethylamine can function as a catalyst for the dehydration of glucose by both intermolecular and intramolecular dehydration process. Diethylamine also can help in doping nitrogen onto graphene in the form of graphitic N, pyridinic N, pyrrolic N, and –C(=O)–NHR. Glucose first forms hydroxymethylfurfural (HMF, 1) which can undergo hydrothermal carbonization in the absence of EDA and HCl (HTC, 2) [46,50]. In the presence of EDA and HCl, nitrogen-doped HTC (HTC with n-doping, 3) is formed. HTC (HTC with n-doping, 3) further undergoes ring closure reaction to form nitrogen-doped GQDs. (4). Tang et al. [40] have found that the size of the GQDs synthesized from glucose, increases exponentially as the growth time increases. However, the size is not elongated. But, in the present case, the shape of the GQDs was spherical until 5 h with an average diameter of ~8 nm. After 6 h of the commencement of the reaction, the GQDs were found to be elongated with the length of 50–100 nm and an average width of 9.34 nm (Figure 1). Due to their elongated shape, it is appropriate to call this structure a graphene quantum pins (GQPs, 5). A difference between GQDs and GQPs can be observed clearly in TEM and AFM images (Figure 1).

The growth of the GQDs and GQPs is shown in Scheme 1. The –OH, –COOH, and –C(=O)– groups in glucose dehydrate under the experimental conditions in the presence of EDA and HCl to form the N– and Cl– doped graphene structure. The growth of GQDs occurs at the edge of the individual graphene sheets. It is interesting to find that after 6 h of reaction, instead of GQDs, GQPs were obtained. This pin like growth could take place in two ways, (i) the spherical GQDs could join together or (ii) there is a continuous growth of the quantum particles in a preferential direction. We presume the second possibility is more plausible, as the first possibility will result in both regular and irregular shapes with voids in between the repeating units. This is supported by the HR-TEM images (Figure 1) with a regular continuity of the graphene sheets in a single direction, without any void space.

**Figure 2:**
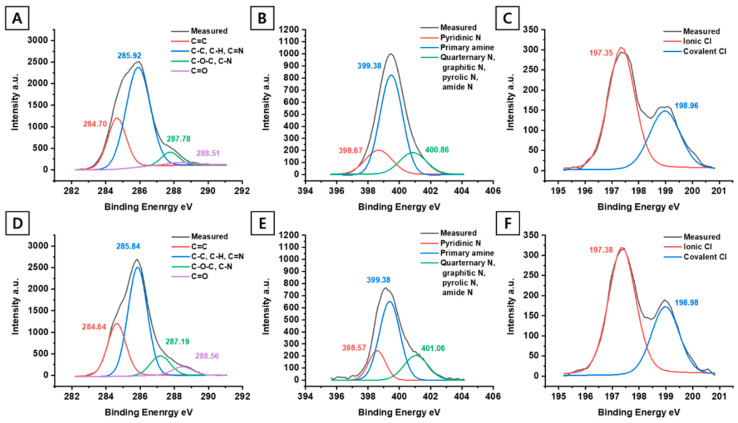
The results of XPS show a clear difference of functional groups on GQPs and GQDs surface. The spectrum of C1s in (**A**) GQPs and (**D**) GQDs can be categorized into 4 groups. The peak intensity is significantly reduced for the COOH group in GQPs with also a visible decrease in the C–O–C intensity. The N1s and Cl2p spectrums of (**B**,**C**) GQPs and (**E**,**F**) GQDs show nitrogen and chloride doping and the presence of tertiary amine.

**Figure 3:**
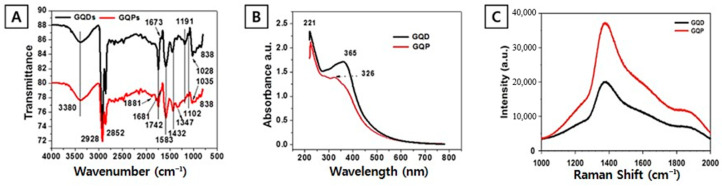
Spectral analysis of obtained GQPs and GQDs. (**A**) FT-IR spectroscopy, (**B**) UV-vis spectroscopy, (**C**) Raman Spectroscopy.

**Figure 4:**
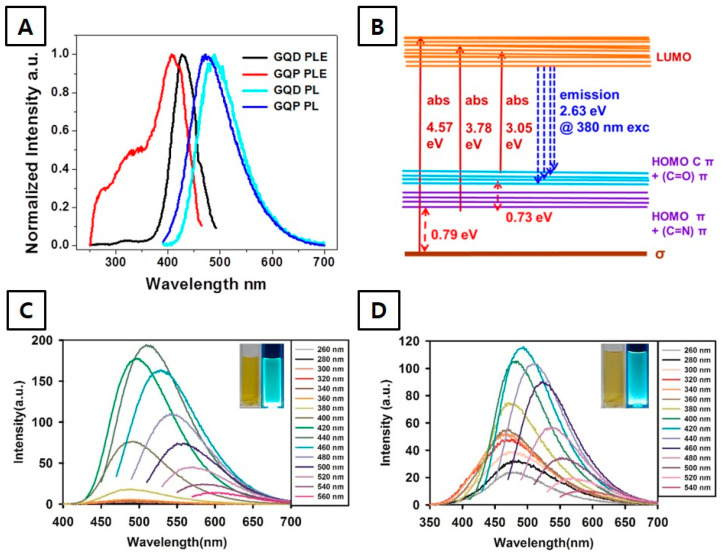
(**A**) Normalized photoluminescence emission (PL), photoluminescence excitation (PLE) spectra of GQPs and GQDs. (**B**) Optical transition for GQPs and typical Fluorescence spectra of obtained (**C**) GQDs and (**D**) GQPs (Inset: Under visible (left) and under 365 nm UV radiation (right)).

The surface functional groups in the GQDs and GQPs include, –OH, –C(=O)–NHR, –C=(O)–OR, epoxy, and –COOH groups. Most of the epoxy groups break and are converted into –OH and –C–NH–(CH_2_)_2_–NH_2_ groups under the reaction conditions. Similarly, COOH groups are converted to –OH and –C(=O)–OR groups. The XPS analysis (Figure 2) and FTIR spectra (Figure 3A) support the presence of these groups. As the emission wavelength (473 nm @ λ_exc_ = 380 nm) is blue-shifted in GQPs compared to GQDs (485 nm @ λ_exc_ = 380 nm) (Figure 4), the bandgap is relatively increased in GQPs compared to GQDs. This can be ascribed to the removal of –COOH groups by –OH and epoxy groups by –OH and –C–NH–(CH_2_)_2_–NH_2_ groups. Consequently, the intensity of emission increases as shown in Figure 4.

The FTIR spectra corroborate the presence of the above functional groups (Figure 3A). The following are the attributes of GQPs FTIR spectra. The –OH group including the –OH form –COOH group appears as a broad peak from 3652 to 3007 cm^−1^ with the center at 3380 cm^−1^ [46,48,49,51]. The N-H stretching of amine and amide usually appears as a broad peak from 3000–3500 cm^−1^. In the present case, the amine and amide N–H stretching peak is merged with the peak at 3380 cm^−1^. The C–H symmetric stretching at 2928 cm^−1^, C–H asymmetric stretching at 2852 cm^−1^ are ascribed to the aliphatic C–H in EDA [52,53] and sp3-defect states in graphene. Vibrations corresponding to C=C–Cl/C=C–O/OH–C=O appear at 1742 cm^−1^ indicating that Cl is doped on to grapheme [51]. Also, the doping of Cl can be observed at 838 cm^−1^ with a small peak, corresponding to C–Cl vibration [46]. C=C peak related to condensed aromatic carbon is observed at 1583 cm^−1^. [46,48,54] C–NH–C (symmetric) and C–NH–C (asymmetric) corresponding to EDA appear at 1191 and 1102 cm^−1^, respectively [46,52,53]. The peak observed at 1035 and 970 cm^−1^ are ascribed to C–O–C (epoxy) group [51] C–N stretching vibration corresponding to the amino group show peak at 1347 cm^−1^ and the small peak 1881 cm^−1^ corresponds to amide C=O stretching. The amide (C–N) stretching appears at 1432 cm^−1^. The FTIR spectrum (Figure 3A) of GQDs and GQPs do not show significant difference with the exception that, the peak at 1742 cm^−1^, has a higher intensity than the GQPs indicating that C=C–Cl/C=C–O/HO–C=O units are present in more number in GQDs. The peak at 1347 cm^−1^ is of low intensity in GQDs compared to GQPs showing the lesser number of amino groups in GQDs. This is supported by the XPS analysis which indicates that nitrogen content in GQPs is higher than GQDs (Figure S1).

The TEM images show the average size of the spherical GQDs to be 8 nm, whereas, the GQPs show clearly their pin shape with the length of GQPs from 50 to 100 nm and average width of 9.34 nm. The inset of Figure 1A,C show the lattice spacing of 0.24 nm corresponding to the [11,20] lattice fringe of graphite [26,40,55,56]. The AFM images (Figure 1B,D) show some aggregation of GQDs and GQPs with maximum heights of 4.92 nm, 68.3 nm, respectively. We presume, the electrostatic attraction due to H–bond between C–Cl, –OH and amine-containing groups result in the stacking of the GQDs and GQPs.

XPS analysis supports the surface functional groups of GQDs and GQPs identified by FTIR spectra and chemical composition of GQDs and GQPs. In full scan XPS spectra (Figure S1A,B) of GQDs and GQPs, three distinct 1s orbital peaks are observed at ~285 eV (C1s), ~399 eV (N1s), and ~531 eV (O1s), indicating that C, N, O are abundant on the surface of GQDs and GQPs. The C1s peak is derived from obtained GQDs and GQPs. The N1s peak is derived from EDA and the reaction of EDA with oxygen-containing functional groups like peroxy and –COOH groups. The O1s peak arises from the oxygen-containing functional groups and SiO2 substrate resulting in a relatively high peak. Also, a small 2p orbital peak is observed at ~197 eV for Cl2p, suggesting that the GQDs and GQPs are doped with chlorine. The atomic concentration is expressed in Table S1. As shown in Table S1, the atomic concentrations are changed after the formation of GQPs. The carbon and nitrogen percentages are increased, and oxygen percentage is decreased in GQPs compared to the GQDs, indicating that the oxygen-containing functionalities are decreased and more of N-containing functional groups are incorporated into the graphene by the reaction of oxygen-containing groups with EDA and other nitrogen-containing functional groups. With the increase in the reaction time from 5 h to 6 h, more number of EDA molecules can react with the oxygen-containing groups to replace the epoxy and COOH groups to –NH(CH_2_)_2_NH_2_ and –C(=O)NH(CH_2_)_2_NH_2_ groups, respectively. These changes and the gradual formation of the ring-forming units in the hetero thermal carbonation process lead to an increase in the size of the GQDs in a particular direction. The nitrogen atoms also occupy the vacancy sites as the pyridinic, graphitic, and pyrrolic nitrogen moieties during the ring closure reaction. The C1s XPS spectrum of GQPs (Figure 2A) and GQBs (Figure 2D) are deconvoluted into four different carbon bond groups (C=C/C–C, C-H, C=N/C–O–C, C–N/C=O). For GQDs peaks at 284.64 eV, 285.84 eV, 287.19 eV, and 288.56 eV correspond to (C=C), (C–C, C–H, C=N), (C–O–C, C–N), (C=O), respectively. For GQPs the peaks at 284.70 eV, 285.92 eV, 287.78 eV and 288.51 eV, correspond to (C=C), (C–C, C–H, C=N), (C–O–C, C–N), (C=O), respectively [49,57]. The peak sites are almost the same for GQDs and GQPs. However, the intensity of the peak is significantly reduced for the COOH group in GQPs with also a visible decrease in the C–O–C (peroxide) peak intensity. The peroxide groups break upon reacting with EDA to form –OH and –C–NH–CH_2_–CH_2_–NH_2_ surface functional groups. It can be presumed that the carboxylic group sites of the GQDs either have been converted to amides or take part in the growth process of GQPs.

The XPS spectra of GQDs and GQPs show nitrogen doping on to the graphene surface (Figure 2B,E) and the relative contents of various elements of GQDs and GQPs in full scan XPS spectrum, Table S2. The N1s XPS spectra of GQPs (Figure 2B) upon deconvolution show peak at 400.86 eV corresponding to graphitic N, pyrrolic N and amide N, 399.38 eV corresponding to primary amine and 398.67 eV corresponding to pyridinic N. Montplaisir and coworkers have reported the binding energy of quaternary amines around 401.16 eV [58,59]. C–N^+^ binding energy of 401.9 eV is reported by Cheng and coworker [60]. Quaternary N at 401.1 eV is reported by Qin’s group [61]. We presume that the peak at 400.86 eV could also be attributed to quaternary alkyl amines derived from EDA. The presence of quaternary amine is confirmed from the Cl2p XPS spectra (Figure 2C). The Cl percentage in GQPs is 1.98%. The Cl2p XPS spectra of GQPs show two peaks at 197.35 eV and 198.96 eV corresponding to salt –NH_3_^+^Cl^−^, and covalent C–Cl. The percentage of salt like Cl of the GQP is 1.19% and that of C–Cl is 0.79%. The salt-like –NH_3_^+^Cl^-^ could also participate in the electrostatic attractions with other groups like, –C(=O) –NH_2_, –NH–CH_2_–CH_2_–NH_2_, –OH, and –COOH, increasing the height of the GQPs as shown by AFM images (Figure 1). The GQDs also show (Figure 2F) similar features in Cl2p spectra with peaks at 197.38 eV and 198.98 eV corresponding to salts like Cl and C–Cl, respectively. In GQDs case the percentage of salt like Cl is 1.11% and that of C–C1 is 0.85%, respectively. The N1s peak of GQDs is deconvoluted (Figure 2E) to get the peaks at 401.06 eV, 399.38 eV, and 398.57 eV, attributable to (i) graphitic N, pyrrolic N and amide N, (ii) primary amine and (iii) pyridinic N, respectively. We also assign 401.06 eV peak to the salt like –NH_3_^+^Cl^-^ nitrogen.

The UV-vis spectral analysis (Figure 3B) of GQDs shows a sharp peak around 221 nm ascribed to the π-π* transition of carbon aromatic sp^2^ domains. The broad band from 300 nm to 350 nm with less intensity is attributed to n-π* transition of C=O bond [46,62]. However, GQPs show a sharper peak around 365 nm due to n-π* transition caused by similar C=O bond with additional influence from increased nitrogen-containing groups supported by XPS analysis. The trapping of excited-state energy by the surface states originated from these groups lead to a strong emission [63]. The GQPs emit around 473 nm when excited by 380 nm wavelength light despite their big sizes and similar to the N-doped GQDs of much smaller size (3 nm) [40]. Raman spectra (Figure 3C) demonstrate the interruption of the orderliness in graphene architecture by doping N and Cl atoms, resulting in the modification and defects in the lattice. The D-band is observed as a broad band peaked at 1350 cm^−1^ and the G-band is almost merged with the D-band. Even though, the small hump is observed around 1600 cm^−1^ which is corresponding to the G band. Usually, D-band indicates the defect site of graphene sheet monolayer, and G-band is related to the vibration of sp^2^ bonded carbon atoms in a 2-dimensional hexagonal lattice. The G-band is highly perturbed due to the doping and introduction of nitrogen-containing functional groups on the graphene surface.

Figure 4A shows the PLE and PL spectra of GQDs and GQPs. The PLE spectra of GQPs show a less intense peak at (i) 273 nm (4.57 eV), (ii) 328 nm (3.78 eV) and a sharper peak at (iii) 406 nm (3.05 eV) with a difference (δE) of 0.79 eV and 0.73 eV between the peak (i) and peak (ii) and peak (ii) and peak (iii), respectively. The transition at 273 nm, 328 nm, and 406 nm can be analyzed (Figure 4B) at the transitions σ → LUMO and (C=C)π (HOMO) (mixed with C=N) → LUMO, (C+C)π (HOMO) (mixed with C=O) → LUMO respectively. δE of 0.79 eV between peaks (i) and (ii) in the PLE spectra indicates that the ground state of the GQPs is of carbene type. Again, the ground state can be considered as a triplet state, as δE is below 1.5 eV [64]. The emission at 472, 482, and 494 nm demonstrate almost similar PLE spectra indicating that the same excited state is involved for the emission to different vibronic levels of the ground state. The optical band gap of GQP is found to be 2.34 eV, which is introduced to the GQPs by the defect states, oxygen, and nitrogen-containing functional groups.

The bandgap and emission wavelength of graphene quantum dots depend on the size [65,66,67], shape [68], defects [69], surface functional groups [70], and the shape of the edges [71]. The density, quantitative, and qualitative nature of the sp^2^ carbons in the graphene nanomaterials are also influenced by the size [68,72]. The zigzag sites and size controls the intrinsic state emission, whereas the surface functional groups comprising oxygen and nitrogen-containing functional groups and defect sites [73] is related to the defect state emissions. The combined effect of both intrinsic state and surface defect state emissions are manifested in the photoluminescence of the graphene nanomaterials. The intrinsic state gives rise to the blue color emission and green luminescence of the graphene nanomaterials is attributed to the surface defect states [51]. In the present case, the HRTEM shows the edges of GQPs are composed mostly of the zigzag arrangement of adjacent carbons along with a few chair arrangements (Figure 1). The zigzag sites, presence of ammine containing functional groups in GQDs and GQPs, bring into blue emission even if the sizes of the GQPs are 50–100 nm. It is interesting that, though the starting materials are the same as that of Yang’s group [46], except the reaction ambiance, the temperature of reaction (Yang’s group 150–200 °C, ours 120 °C) and purification process, we obtained blue emission (472 nm for GQP excited at 380 nm) with the similar excitation wavelength despite the much bigger size (Yang’s group: 5–25 nm), whereas the GQDs obtained by Yang’s group were emitting green light at 524 nm with the excitation wavelength of 375 nm. We presume, the relatively low reaction temperature of 120 °C in the present case compared to the reaction temperature of 150–200 °C of Yang’s group, allowed the building block to bunch up to integrate into bigger particles. It is to be noted that, after 5 h of reaction GQDs of average size 8 nm were obtained, whereas, after another hour much bigger GQPs (50–100 nm) were formed. Therefore, a one-hour extra reaction time was enough to streamline the building units for convergence. We presume, the ring-forming building blocks saturated the reaction medium after 6 h of reaction only to combine to form GQPs. It is interesting to find that the PLE spectrum of GQDs (Figure 4A) is different from that of GQPs. It has an intense peak at 426 nm with a shoulder at 462 nm. The shoulder at 462 nm acquires more intensity as the emission wavelength is bathochromically shifted (Figure 4A).

**Figure 5:**
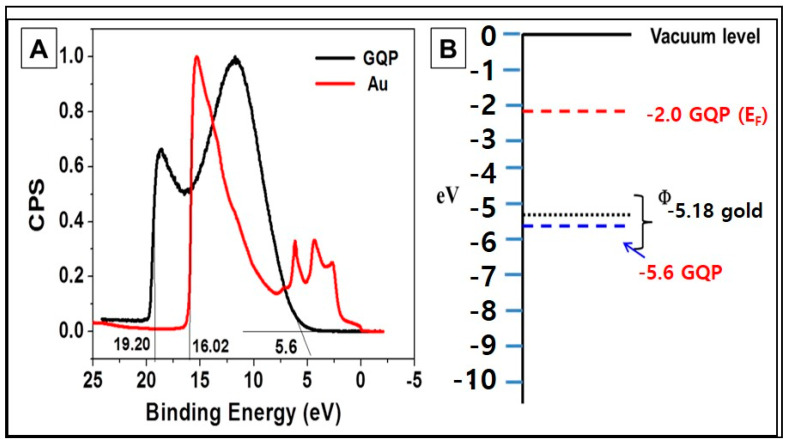
(**A**) Ultraviolet photoelectron spectroscopy (UPS) spectra (**B**) Fermi energy, a work function of GQPs (taking gold as standard).

The photoluminescence (PL) spectra of the GQDs and GQPs (Figure 4C,D) exhibit blue emission under 360 nm UV radiation. The emission peak does not shift appreciably with the increase of excitation wavelength from 260 nm to 420 nm in the case of GQDs. However, upon increasing the excitation wavelength from 440 nm to 540 nm, the emission peaks are shifted from 508 nm to 568 nm by an interval of 13–20 nm. For GQP, the emission peak did not show the shift with the increase of excitation wavelength from 260 nm to 360 nm. However, when the excitation wavelength increases from 380 nm to 540 nm, the emission peaks are bathochromically shifted from 472 nm to 533 nm by an interval of 10–17 nm. The difference in emission wavelength can be derived from the difference of the composition of surface states [46]. The Fermi energy (EF) and work function (WF) of the GQPs were found to be −5.6 and −2.0 eV, respectively from the ultraviolet photoelectron spectroscopy (UPS) spectra (Figure 5A,B) considering gold EF = 0. The band from 5.0 eV to 16.5 eV is ascribed to the hybridization of C2p with O2p and possibly N2p in the neighboring molecules or GQPs. The origin of the secondary edge from 18.5 to 19.9 eV could be due to the presence of ionic chloride in a disorderly insulator like structure inducing a different local work function.

### 3.2. Cytotoxicity and ROS Measurement

**Figure 6:**
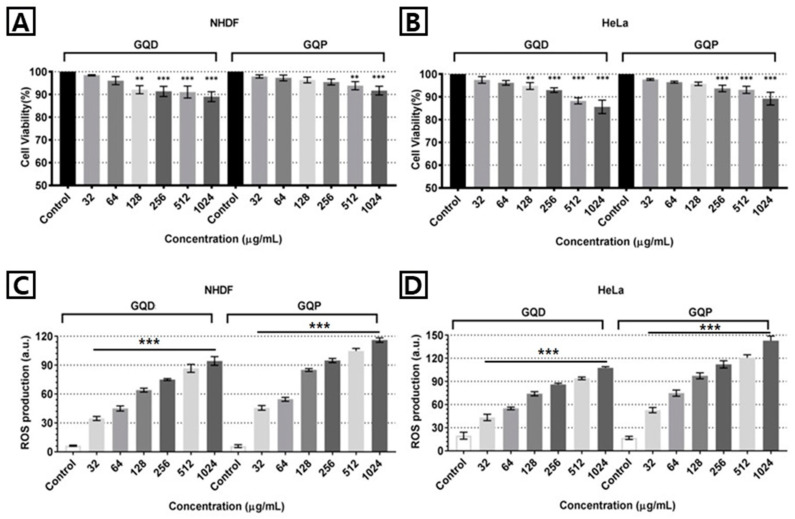
Toxicity of GQDs and GQPs in (**A**) normal human dermal fibroblast (NHDF) and (**B**) HeLa. Cell viability of GQDs and GQPs measured using CCK-8 assay. Experiments were repeated thrice with the triplicate, and significant difference between control and test samples are marked with ** (*p* < 0.01), and *** (*p* < 0.001). ROS generation level of GQDs and GQPs in (**C**) NHDF and (**D**) HeLa. The level of GQDs and GQPs measured using DCFH-DA assay. Experiments were repeated thrice with the triplicate, and a significant difference between control and test samples are satisfied in all test concentrations.

To investigate the potential of GQPs for biological applications such as bio-imaging, delivery carrier, the cytotoxicity of GQPs was evaluated using two different cell lines—Normal human dermal fibroblast (NHDF) and human cervical cancer cell (HeLa) with CCK-8 assay. As shown in Figure 6A,B, upon the treatment of GQDs and GQPs, the cytotoxicity exhibited a dose-dependent increase. A significant cell viability decrease was observed at the concentration of GQDs ranging from 64 μg/mL to 128 μg/mL compared to that of control (*p* < 0.05). In the case of GQPs, it was statistically different from the control when the concentration of GQPs ranges from 256 μg/mL to 512 μg/mL (*p* < 0.05). Besides, the cell viability decreases of GQDs for HeLa cells had a statistically valid value in a similar range with NHDF. But the cell viability of GQPs for the HeLa cell was statistically significant at the range of concentration between 128 μg/mL and 256 μg/mL. For NHDF, the cell viability of GQDs and GQPs showed high cell viability near 90% at the highest concentration. In the case of HeLa cells, it reduced slightly as compared with NHDF, but it still showed high cell viability. These results indicate that the good biocompatibility of GQPs is not limited to specific cells. The previous studies reported that graphene oxide (GO) showed higher cytotoxicity than GQDs [74,75,76] and also suggested that the small size of GQDs is the reason why GQDs have lower cytotoxicity than GO. However, in the case of GQPs, the reason why GQPs showed low cytotoxicity despite their large size might be that the functional groups of GQPs led to less damage to the cells.

To determine the cause of cytotoxicity, DCFH-DA assay was carried out with assuming that ROS generation affects the cell damage [77]. Figure 6C,D clearly show that the treatment of GQDs and GQPs with NHDF and HeLa caused an increased ROS generation compared to the control cell. Therefore, the cytotoxicity observed in NHDF and HeLa cells can be caused by the ROS generation induced by GQDs and GQPs. According to the previous studies, graphene oxide could induce ROS generation inner cell membrane, whereas GQDs caused lower ROS generation of cells than graphene oxides [47,74]. Corresponding to the previous studies, our results also demonstrate the ROS generation derived from graphene quantum material. Despite the bigger size of GQPs, ROS generation by both GQDs and GQPs is observed by a similar amount at the same concentration. These results from cell viability study and ROS generation measurement suggest that these two materials are highly biocompatible at the cellular level.

### 3.3. Cellular Response

The change of biophysical and biomechanical properties of NHDF and HeLa cells can be described as the cellular response to GQDs and GQPs. Several studies explained the interaction between the cellular response upon the treatment of nanomaterials and stem cell differentiation. These studies utilized Bio-AFM to investigate the biophysical and biomechanical changes in differentiating cells and nanomaterials treated cells [41,48,78,79]. The change in biophysical (morphology, cell height, RMS roughness, and cell spreading area) and the biomechanical (Cell stiffness; Young’s modulus) properties were reported. In this study, the cells were treated with GQDs and GQPs (512 μg/mL) for 24 h and then investigated using Bio-AFM. The morphology images of the cells were taken at three magnifications (100 μm × 100 μm, 40 μm × 40 μm, 10 μm × 10 μm).

**Figure 7:**
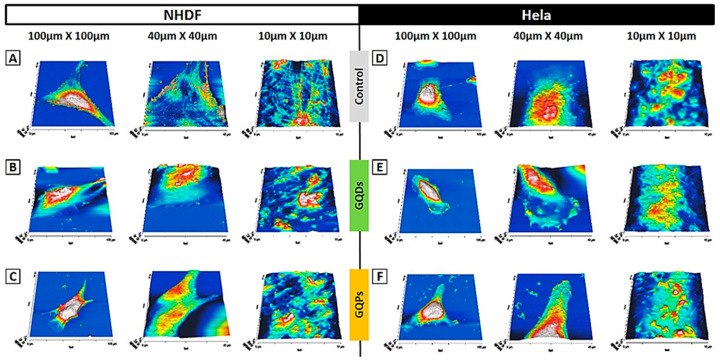
AFM image of cells after treatment of GQDs and GQPs. AFM 3D images of control cells; (**A**) NHDF, (**D**) HeLa. In the case of NHDF, compared to the control, the cell surface became much rougher when the cells were treated with (**B**) GQDs and broader when the cells were treated with (**C**) GQPs. The pores and blebs, which indicate cell apoptosis, were found to be larger upon cell treatment with GQDs and GQPs, as compared to the control group. In the case of HeLa, no specific change in cell shape was observed, but the blebs on the surface were more obvious after treatment with (**E**) GQDs than after treatment with (**F**) GQPs.

**Figure 8:**
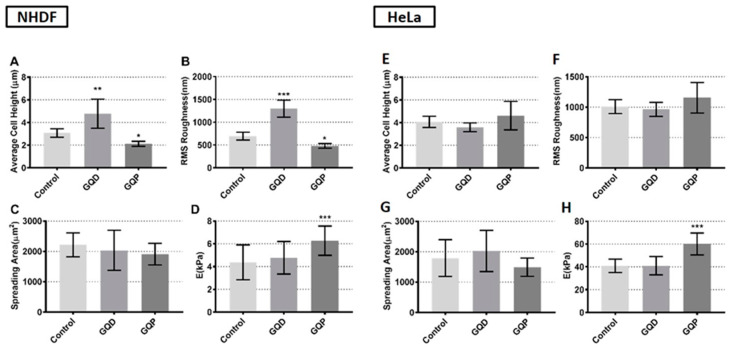
Quantitative analysis of cellular responses upon treatment with GQDs and GQPs in NHDF and HeLa. Graphs show (**A**,**E**) average cell height, (**B**,**F**) RMS roughness, (**C**,**G**) cell spreading area and (**D**,**H**) stiffness of cells, respectively. Experiments were repeated thrice with triplicate and significant differences between control and test samples are marked with * (*p* < 0.05), ** (*p* < 0.01), and *** (*p* < 0.001).

None of the cells were found to show any significant change in cell morphology except blebs. Filopodia, lamellipodia, invadopodia were observed in all groups. The overall shape of cells did not have any distinctive changes except the cell surface. The changes in cell surface were obvious after GQDs and GQPs treatment. GQDs show some blebs on the surface in the case of NHDF, indicating apoptosis (Figure 7B), compared to the control (Figure 7A). Treatment with GQPs shows a broader surface than GQDs treatment (Figure 7C). In the case of HeLa cells, the control and treatment with GQDs show many blebs on the surface compared to GQPs. (Figure 7D–F) The SEM images of cells supported the results of AFM images (Figure S2A–F). Quantitative analysis of cellular response can demonstrate the difference in response following the treatment of GQDs and GQPs (Figure 8A–H). The cellular response was categorized into 4 groups which are average cell height, RMS roughness, cell spreading area, and Young’s modulus. Among these variables, the valid statistical variables are the average cell height, RMS roughness after GQDs treatment in NHDF. In the case of GQPs treatment, the average cell height, RMS roughness, and Young’s modulus have a statistically valid value. The cell average height was increased to 4.78 μm from 3.07 μm after GQDs treatment, but it was reduced to 2.11 μm from 3.07 μm after GQPs treatment as compared to the control group. The RMS roughness was increased to 1297.83 nm from 694.7 nm after GQDs treatment, but it was reduced to 480 nm from 694.7 nm after GQPs treatment. Furthermore, Young’s modulus is increased to 6.27 kPa from 4.38 kPa after GQPs treatment. In HeLa cells, the valid statistical variable is only the change in Young’s modulus after the treatment with GQPs. The Young’s modulus was increased to 60.06 kPa from 40.93 kPa, as compared to the control group, after GQPs treatment. From these results, we infer that in the case of NHDF, GQDs deposit strongly on the cell surface or penetrate the cells, but it does not generate mechanical tension/stress. But, when GQPs adhere to the cell surface, it does not change the RMS roughness due to their size. Also, while GQPs translocate into the cells, mechanical tension/stress in the cytoplasm is generated. However, in HeLa cells, although GQDs deposit on the cell surface or penetrate the cells, GQDs do not affect the biophysical and biomechanical properties. It can be inferred that GQDs treatment does not trigger structural reorganization. In the case of GQPs, while GQPs translocate into the cell, they change Young’s modulus, indicating the reorganization of the cytoskeleton. The details of the variables are shown in Table S3. From these valid values, it can be assumed that GQPs affect the cellular biophysical and biomechanical properties and the treatment with GQPs can block physically the cell death from the chemical attack such as ROS.

### 3.4. Bio-Imaging and Cellular Distribution

**Figure 9:**
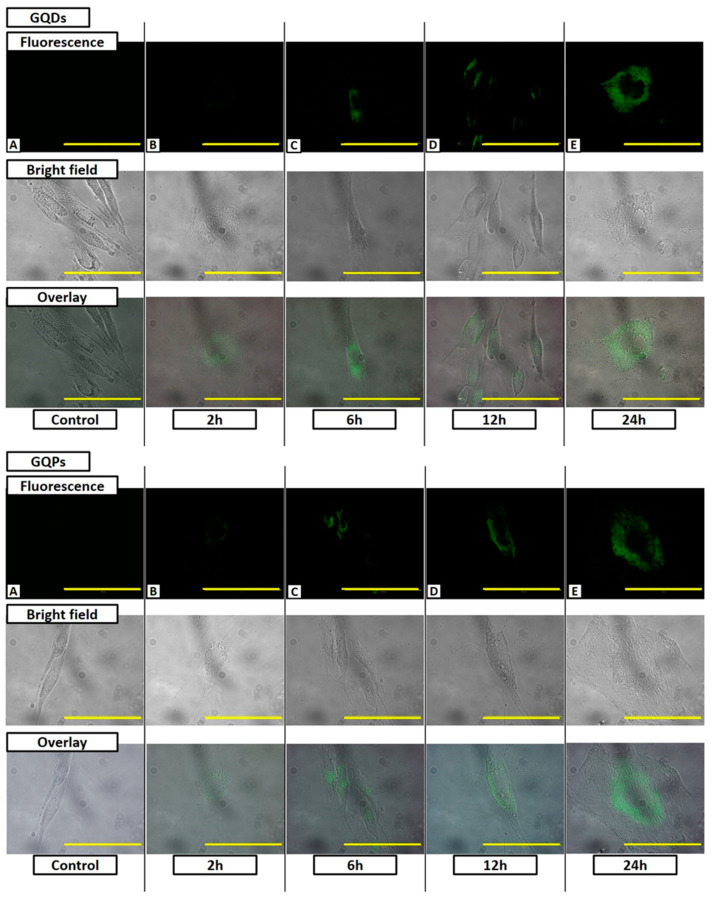
Bio-imaging of GQDs and GQPs in NHDF. Fluorescence emission of both GQDs increased with increasing incubation time. Each scale bar (yellow bar) was 100 μm.

Bio-imaging was carried out with a fluorescence microscope for observing the fluorescence image of the cell. Images were taken according to the incubation time. The green color results from the fluorescence of GQDs and GQPs, since the filter cube (GFP, excitation: 470 nm/emission: 530 nm) limits the emission color of fluorescence to green color. The image types were categorized into a fluorescence, bright-field, and overlay image. In a bio-imaging analysis, the fluorescence behaviors of GQDs and GQPs after treatment to NHDF and HeLa were different. In NHDF, the fluorescence emission of the GQDs and GQPs is thronged around the nucleus region (Figure 9). On the other hand, in HeLa, the fluorescence emission of GQDs and GQPs spread from the nucleus region throughout the cells (Figure S3).

**Figure 10:**
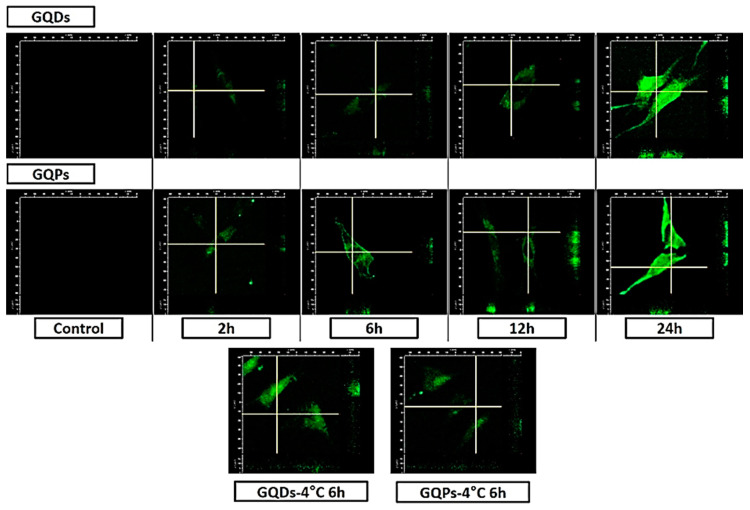
3D reconstructed confocal images for analysis of cellular distribution of GQDs and GQPs in NHDF. The fluorescence emission was present only in cytoplasm except nucleus until 12 h. After 24 h, it was observed in the whole cell. The two images at the bottom of figure were the 3D reconstructed confocal images of blocking endocytosis at low temperature. X and *Y*-axis scale are 100 μm and the yellow line indicates the cross-section of confocal image.

Cellular distribution was monitored with a laser scanning confocal microscope for determining and elucidating GQDs and GQPs internalization. Images were transformed into 3D-reconstructed confocal images. X and *Y*-axis scale are 100 μm each and the yellow line indicates the cross-section of confocal image. In the case of normal culture conditions, the cellular distribution of NHDF can be seen in Figure 10. The fluorescence emission intensity increased with increasing incubation time, which indicates that the cellular uptake of GQDs and GQPs is time-dependent, but the fluorescence emission was not shown in the control group. Also, a 3D-reconstructed confocal image of cells treated with GQDs and GQPs showed that the fluorescence emission was present only in cytoplasm except for the nucleus until 12 h. Fluorescence emission was observed in the whole-cell after 24 h. The cellular distribution of HeLa was similar to NHDF, which increase the fluorescence emission intensity with increasing incubation time (Figure S4). Also, the fluorescence emission was not observed in the control group. The 3D-reconstructed confocal images of blocking endocytosis were shown in the bottom of Figure 10 and Figure S4. These images reveal that the pathway of the cellular uptake of GQDs, GQPs does not follow an energy-dependent mechanism such as endocytosis, but possibly direct penetration into the cell membrane corresponding with previous studies which demonstrated about the mechanism of cellular uptake of graphene oxide and carbon nanotube [80,81,82,83].

Several studies have found that GQDs can enter the cell nucleus, [84,85] whereas some studies have demonstrated that GQDs are distributed in the cytoplasm [54,73,86,87]. The different functional groups on the graphene nanomaterials and cell type can cause different cellular distribution. From our fluorescence image, the distribution site of GQDs, GQPs were not specific. In NHDF, the fluorescence of GQDs and GQPs was not observed in the nucleus until 12 h after treatment, but it shows the possibility of distribution in the whole-cell after 24 h. Interestingly, in HeLa, the cellular distribution was not specific to any part. These results suggest that GQPs can be used as a bio-imaging agent like GQDs.

## 4. Conclusions

We synthesized a new kind of graphene quantum material called “graphene quantum pin” via a bottom-up approach. GQPs and GQDs isolated from the same reaction mixture have different morphologies. The GQPs show sharper absorption compared to GQDs. Despite the bigger size of the GQPs compared to GQDs, the emission from GQPs is blue shifted. The blue shift in the emission is attributed to the presence of a greater number of –C(=O)–NH–CH_2_–CH_2_–NH_2_ and –NH–CH_2_–CH_2_–NH_2_ groups on the surface. In the cytotoxicity study, GQPs show higher biocompatibility than GQDs. The pattern of fluorescence emission of GQPs is similar to that of GQDs in bio-imaging and cellular distribution. The change in fluorescence emission intensity with incubation time demonstrate a time-dependent cellular uptake of GQDs, GQPs by the cells. In the case of blocking endocytosis, both samples show direct membrane penetration. In cellular response studies, GQDs were found to get deposited and translocated in NHDF cells without changing the cell stiffness, whereas GQPs were translocated and increased the cell stiffness. GQDs and GQPs were also deposited and translocated into HeLa, but there was only the change of cell stiffness after GQPs treatment. These results suggest that GQPs can be used as a potential bio-imaging agent as GQDs as fluorescence is concerned. The synthesized GQDs and GQPs are potential materials for bioimaging, drug delivery, biosensing and other biomedical applications. Therefore, as a next step we intend to continue the experiment in the biosensing application.

## Supplementary Materials

The following are available online at https://www.mdpi.com/2079-4991/10/12/2550/s1, 1: Full scan XPS spectrum of (**A**) GQPs and (**B**) GQDs, Figure S2: SEM image of cells after treatment of GQDs and GQPs, Figure S3: Bio-imaging of GQDs and GQPs in HeLa. Fluorescence emission of both GQDs increased with increasing incubation time, Figure S4: 3D reconstructed confocal images for analysis of cellular distribution of GQDs and GQPs in HeLa. The fluorescence emission was present in whole cell. The two images at the bottom of figure were the 3D reconstructed confocal images of blocking endocytosis at low temperature, Table S1: The relative contents of various elements of GQDs and GQPs in full scan XPS spectrum, Table S2: The relative contents of various bonds of GQDs and GQPs in C1s XPS spectrum, Table S3: Detail value of cellular response (Avg: average, Std: Standard deviation).
