# Supplementary material for: Cytotoxicity and Bioimaging Study for NHDF and HeLa Cell Lines by Using Graphene Quantum Pins
Source: Nanomaterials (Basel). 2020 Dec 18;10(12):2550. doi: 10.3390/nano10122550 (PMC7766917; doi:10.3390/nano10122550)
Supplement: Supplementary file 1 [file nanomaterials-10-02550-s001.pdf]

Supplementary materials

Cytotoxicity and Bioimaging Study for NHDF and HeLa Cell Lines by Using Graphene Quantum Pins

Seong-Beom Jeon [1,2], Monica Samal [3], Saravanan Govindaraju [1], Rupasree Ragini Das [1] and Kyusik Yun [1,*]

[1] Department of Bionanotechnology, Gachon University, Seongnam 13120, Korea; chaoshit@gc.gachon.ac.kr (S.-B.J.); biovijaysaran@gmail.com (S.G.); rupasree.ragini@gmail.com (R.R.D.);

[2] School of environmental and Science engineering, Gwangju Institute of Science and Technology (GIST), Gwangju 61005, Korea; sbjeon0430@gist.ac.kr (S.-B.J.)

[3] Department of Material Science and Engineering, North Carolina State University, Raleigh, NC 27695, USA; ms.phd.sci@gmail.com (M.S.)

* Correspondence: ykyusik@gachon.ac.kr (K.S.Y)

Supplementary Methods:

Synthesis of GQDs and GQPs

GQPs were synthesized by carbonization and dehydration of glucose via the modified method of previous report by Yang's group. In a 250 mL round bottom flask, 10 g of D-(+)-glucose (Sigma Aldrich, St. Louis, USA) were taken and dissolved in 150 mL of DI water under water bath sonication (JAC Ultrasonic 2010) for 15 min until full dissolution. Then, 4 mL of Ethylene diamine (EDA; Sigma Aldrich) and 2 mL of HCl (Sigma Aldrich) added into the flaks. The flask was then connected to a condenser maintaining the temperature at −10 °C, followed by gradual heating of the solution to 120 °C by heating mantle for 6 h. After reaction, the final solution was dialyzed by a dialysis membrane (Spectrum Lab, MWCO: 2000) for 2 d to obtained GQPs. Meanwhile, every 12 h the outside solution was collected and dialyzed by dialysis membrane (Spectrum Lab MWCO: 1000) for 2 d. Every 12 h, the solution was making it concentrated by using rotary evaporator to get GQDs. After purification process, the GQDs and GQPs solutions were freeze-dried perfectly at −50 °C for 3 d.

Characterization

Morphology (AFM, HR-TEM)

The morphology and topography of synthesized GQDs and GQPs were characterized by using HR-TEM (High Resolution Transmission Electron Microscopy, Tecnai, F20, FEI, Oregon, USA) and AFM (Atomic Force Microscopy). AFM analysis was carried out with Bio-AFM (JPK instrument, Bruker Co., Billerica, USA) mounted on inverted optical microscope (Nikon Eclipse Ti-U, Tokyo, Japan). This instrument was isolated totally from other light and noise during the measurement. The probe used for measurement was silicon nitride AFM probes (Budget sensor, Sofia, Bulgaria). Si wafer was treated with O_2 plasma and sample solution were dropped onto Si wafer and dried perfectly. AFM study was carried out in air contact mode. TEM images were taken by High-Resolution TEM. For sample preparation, sample solutions were dropped onto copper grid and dried perfectly.

Spectral analysis (UV, FT-IR, XPS, UPS, Raman, Fluorescence spectroscopy)

Spectral analysis of GQDs and GQPs were done by using UV-vis spectroscopy (V-730, JASCO,

Tokyo, JApan), FT-IR (Jasco, FT/IR 4600), XPS and UPS (PHI 5000 versa Probe, ULVAC PHI, Kanagawa, Japan), Raman spectroscopy (Renishaw, UK), Fluorescence spectroscopy (Cary Eclipse Fluorescence spectrometer, Agilent, California, USA). In UV-vis spectral measurement, the quartz cuvette was used and the concentration of samples were 512 μg/ml. For measuring FT-IR spectrum, the surface of quartz glass was cleaned by using ethanol, DI water, in turn. And then, the sample was smeared and was measured in the range of 500 cm^{-1} to 4000 cm^{-1}. XPS is monitored with thin film of samples on 1 cm × 1 cm Si wafer. For Raman spectroscopy, one drop of each sample solution was dropped onto slide glass. The photon energy of the UPS light source (He(I) radiation) was 21.2 eV at a 90 degree tilt, light off, with a pass energy of 0.585 eV and at a bias of -7 eV. During calculation we have kept the bias energy single. A laser of 785 nm was used to measure Raman signal in the range of 1000 cm^{-1} to 2000 cm^{-1}. For the photoluminescence (PL) measurements, excitation wavelengths from 260 to 540 nm in 20 nm intervals were used.

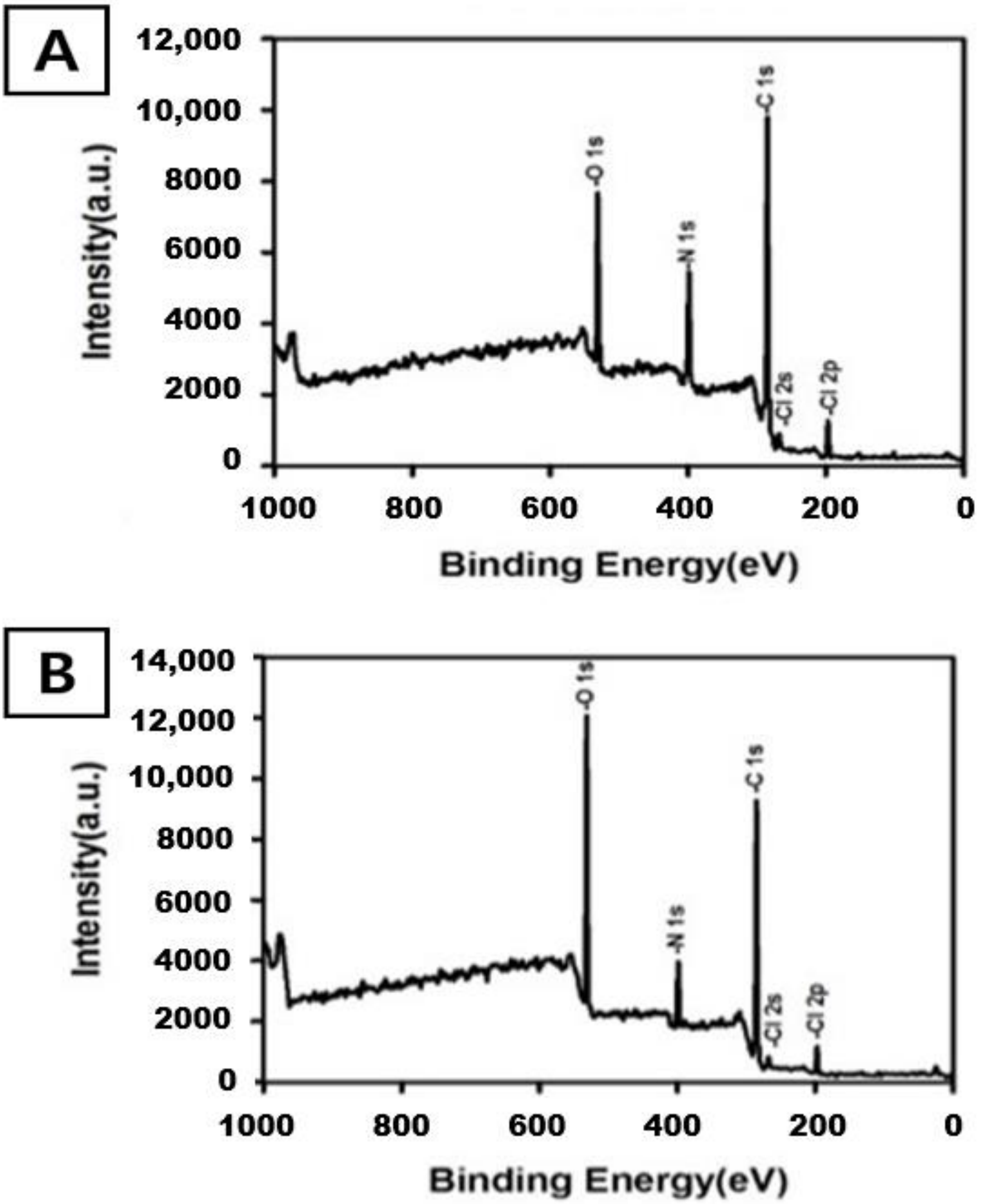

Figure. S1. Full scan XPS spectrum of (A) GQPs and (B) GQDs

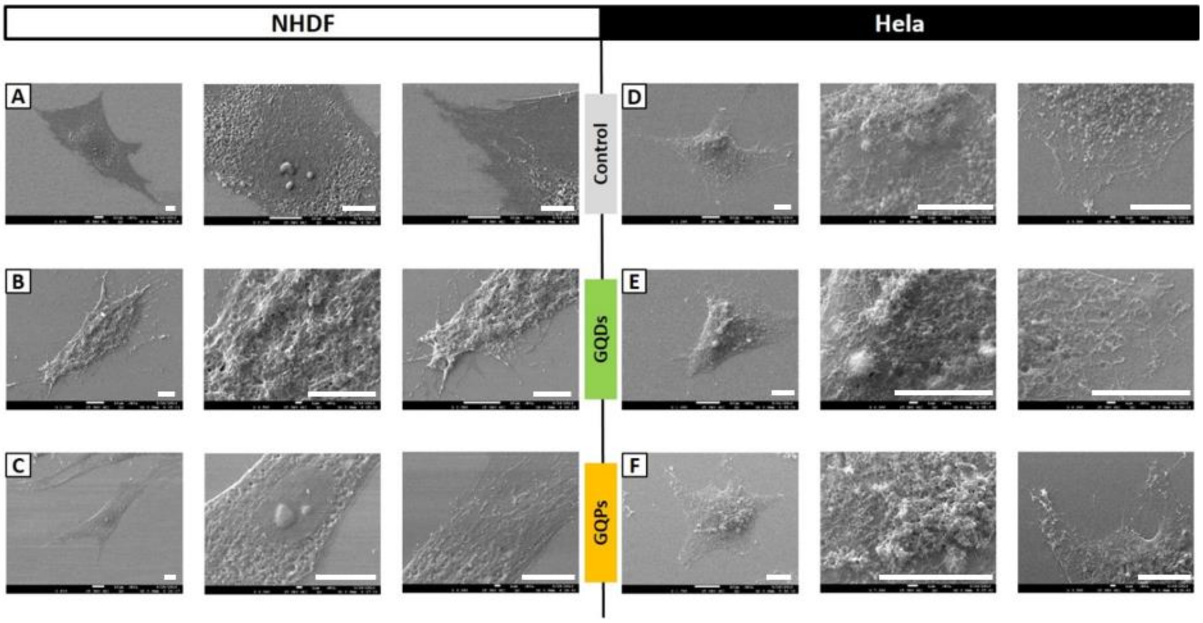

Figure. S2. SEM image of cells after treatment of GQDs and GQPs.

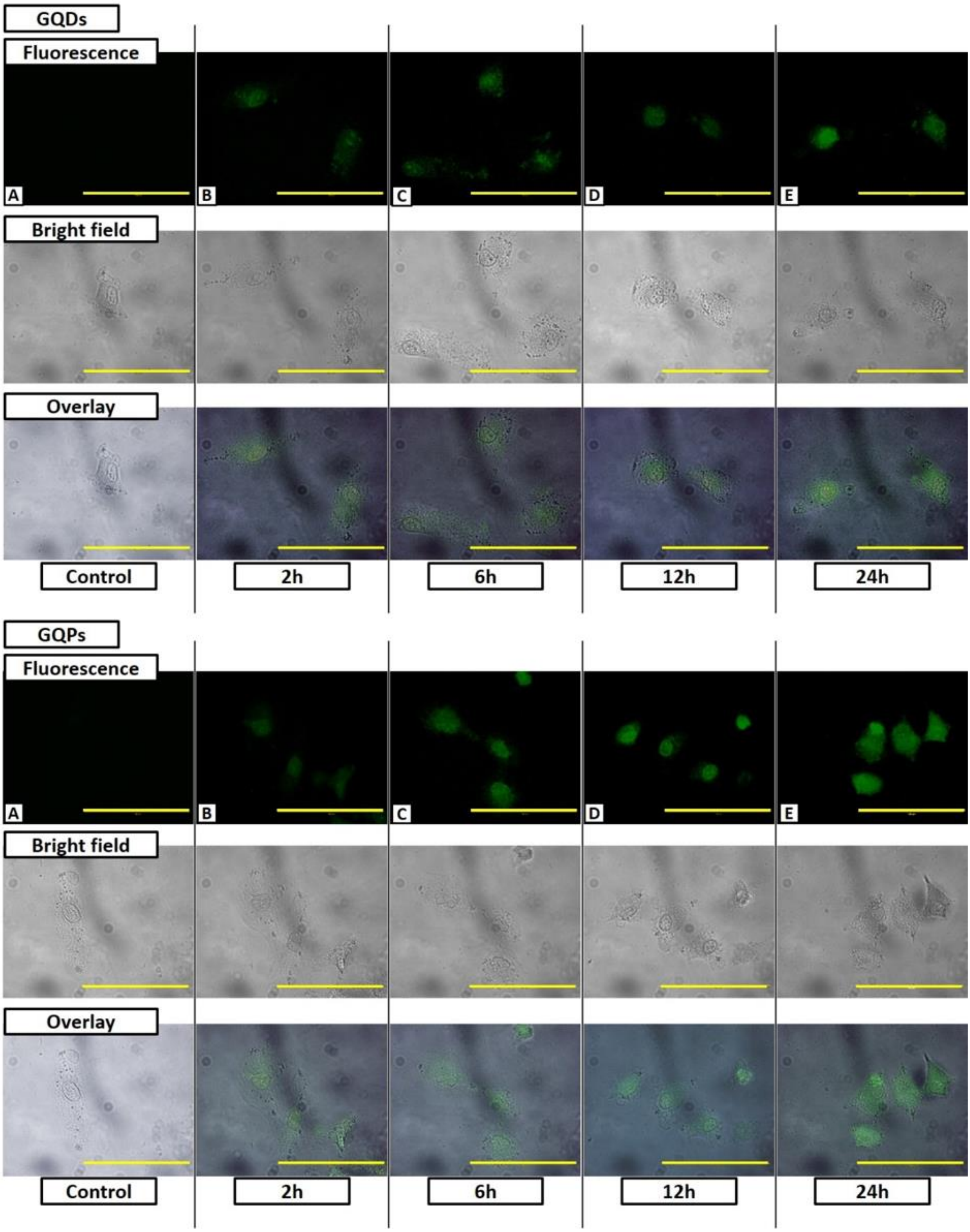

Figure. S3. Bio-imaging of GQDs and GQPs in HeLa. Fluorescence emission of both GQDs increased with increasing incubation time.

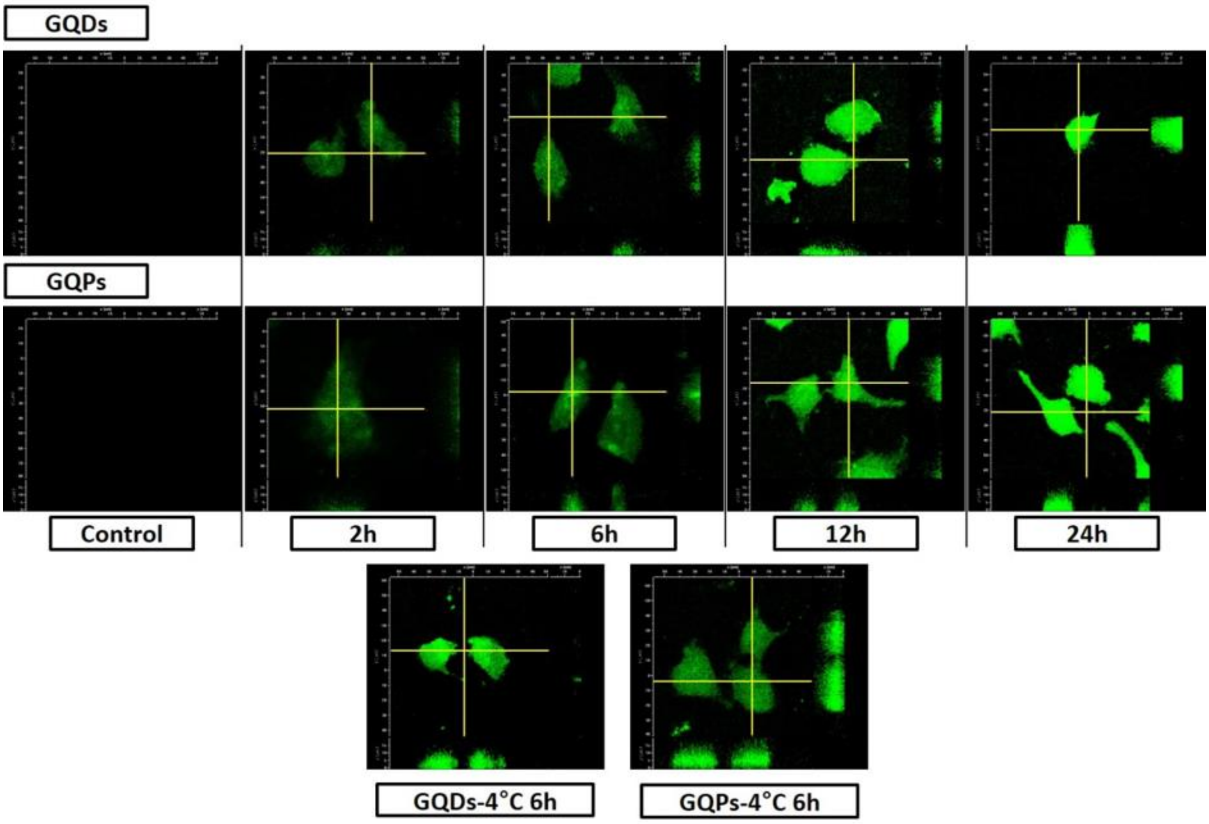

Figure. S4. 3D reconstructed confocal images for analysis of cellular distribution of GQDs and GQPs in HeLa. The fluorescence emission was present in whole cell. The two images at the bottom of figure were the 3D reconstructed confocal images of blocking endocytosis at low temperature.

Table S1. The relative contents of various elements of GQDs and GQPs in full scan XPS spectrum.

	GQDs	GQPs
**Elements**	**Relative content(at. %)**	**Relative content (at. %)**
**C 1s**	**63.03**	**68.24**
**O 1s**	**24.55**	**15.58**
**N 1s**	**10.47**	**14.20**
**Cl 2p**	**1.96**	**1.98**

Table S2. The relative contents of various bonds of GQDs and GQPs in C1s XPS sp ectrum

GQDs			GQPs		
Bond	Binding energy (eV)	Relative content (at.%)	Bond	Binding energy (eV)	Relative content (at.%)
C=C	284.64	27.85	C=C	284.70	28.27
C─C, C─H, C=N	285.84	56.91	C–C, C–H, C=N	285.92	63.92
C–O–C, C–N	287.19	10.56	C–O–C, C-N	287.78	5.90
C=O	288.56	4.68	C=O	288.51	1.92

Table S3. Detail value of cellular response (Avg : average, Std : Standard deviation)

	NHDF			HeLa		
	Control	GQDs	GQPs	Control	GQDs	GQPs
Cell height (µm)	3.07	4.78	2.112	4.06	3.58	4.61
RMS roughness (nm)	694.7	1297.83	480.06	1008.67	963.86	1153.81
Cell spreading area (μm^2)	2217.78	2037.07	1909.81	1798.12	2025.82	1492.21
Cell stiffness (kPa)	4.38	4.77	6.27	40.93	41.04	60.09
